# Draft genome sequence of a resistant diesel fuel degrader *Rhodococcus ruber* IEGM 442

**DOI:** 10.1128/mra.00255-26

**Published:** 2026-05-18

**Authors:** Liliia Komarova, Anastasiia Krivoruchko, Maria Kuyukina, Irina Ivshina

**Affiliations:** 1 Perm Federal Research Center, Perm, Russia; 2 Perm State University 64949, Perm, Russia; Wellesley College, Wellesley, Massachusetts, USA

**Keywords:** bioremediation, diesel fuel, *Rhodococcus ruber*, draft genome

## Abstract

*Rhodococcus ruber* IEGM 442 was isolated from snow at an oilfield. It shows a pronounced diesel fuel-degrading activity. We report a draft genome sequence of this strain obtained using the Illumina technology. It can be used to reveal genetic mechanisms of diesel fuel biodegradation by *Rhodococcus* bacteria.

## ANNOUNCEMENT

*Rhodococcus ruber* IEGM 442 survives in the presence of 16-32 vol. % diesel fuel and degrades 42% diesel fuel at its concentration of 8 vol. % in 8 days (1). Therefore, it is promising for bioremediation. It was isolated from the oilfield snow in the Perm region, Russia, taken aseptically. After melting, the 100 μL of undiluted sample was spread on the mineral agar K and incubated in atmosphere propane:air 1:5 (http://www.iegmcol.ru/medium/med05.html) at 28°C (2). A pure culture was obtained after sequential transfers of the target orange colony on the same fresh medium. The IEGM 442 strain was identified on the basis of its morphology, enzymatic activities determined according to Bergey’s Manual (3), and a positive result in the species-specific polymerase chain reaction performed using Taq DNA polymerase (Evrogen, Moscow, Russia), primers Ru1 5′-GTCTAATACCGGATAGGACCTCGGGA-3′ and Ru2 5′- TACCGTCACTTGCGCTTCGTCGGTAC-3′ to the 16S rRNA gene (4). The strain was deposited in the Regional Specialized Collection of Alkanotrophic Microorganisms (acronym IEGM, WDCM #768, http://www.iegmcol.ru/strains/rhodoc/ruber/r_ruber442.html).

Cells were recovered from a lyophilized culture after storage for 5 months and grown in the Luria-Bertani broth at 160 rpm at 28°C for 28 h. DNA was isolated from a pure culture using the Quick-DNA Fungal/Bacterial Microprep Kit (Zymo Research, Irvine, CA, USA) following the manufacturer’s protocol and quantified using a Qubit 4 fluorometer (Thermo Fisher Scientific, Waltham, MA, USA). The quality was assessed by 1% agarose gel electrophoresis. A total of 20.944 μg DNA was obtained, and 100 ng was used for sequencing. A paired-end library (2 × 100 nucleotides [nt]) was produced using Illumina DNA Prep and sequenced on an Illumina NovaSeq 6000 instrument (Illumina, Inc., San Diego, CA, USA). The library’s quality was estimated using a fluorescence analysis and a 2100 Bioanalyzer (Agilent Technologies, Santa Clara, CA, USA). A total number of 29,709,289 paired-end reads was obtained. Demultiplexing was performed with Illumina bcl2fastq (v2.20), and adapter trimming was conducted using Skewer (v0.2.2) (5). The quality of the FASTQ files was analyzed with FastQC (version 0.11.5-cegat) (6). The data were assembled with SPAdes v. 3.15.4 (7) at the contig level. The assembly consisted of 114 contigs with a total sequence length of 5,823,403 bp, N50 value of 145,262 bp, guanine-cytosine content of 70.5%, coverage of 435×, completeness of 99.33%, and contamination of 0.4%.

Average nucleotide identity (ANI) and digital DNA/DNA hybridization (dDDH) were calculated using ANI Calculator (https://www.ezbiocloud.net/tools/ani) (8), Genome-to-Genome Distance Calculator 3.0 (https://ggdc.dsmz.de/ggdc.php#) (9), and Type (Strain) Genome Server (https://tygs.dsmz.de/) (10). The highest OrthoANIu and dDDH (formula d_4_) values of 99.18 and 92.5%, respectively, were obtained for *R. ruber* NBRC 15591^T^ (NCBI RefSeq assembly acc. no GCF_001894945.1/). The annotation of coding sequences (CDS) was performed using PGAP 6.9 (11). Default parameters were used for all software.

A total of 5,358 CDS, 5,277 CDS with protein, and 58 RNAs were found. The 35 CDS coded for monooxygenases, including two methane (propane) oxidizing complexes (2), two alkane 1-monooxygenases and 24 cytochromes P450, 19 CDS coded for alcohol dehydrogenases, and 33 CDS coded for aldehyde dehydrogenases.

## Data Availability

This Whole-Genome Shotgun project has been deposited in DDBJ/ENA/GenBank under accession numbers JAPWIV010000001–JAPWIV010000114. The version described in this paper is the first version available as assembly GCF_027270695.1. Raw sequence reads were deposited in the Sequence Read Archive with accession number SRR34674032 under BioSample accession number SAMN32240022 and BioProject accession number PRJNA783162.
